# A case report of fractured guidewire removal by rotational atherectomy

**DOI:** 10.1186/s13019-021-01724-3

**Published:** 2021-11-27

**Authors:** Xu Wang, Jian Ye, Jun-Qing Gao, Zong-Jun Liu

**Affiliations:** Department of Cardiology, Putuo Hospital, Shanghai University of Traditional Chinese Medicine, Shanghai, 200062 People’s Republic of China

**Keywords:** Rotational atherectomy, Percutaneous coronary intervention, Fractured guidewire, Drug-eluting stent

## Abstract

**Background:**

Fractures occur in association with manipulation and because of the complexity of the coronary artery, and they can cause a series of serious complications, such as myocardial infarction and secondary thrombosis. Common treatments for fractured guidewires include conservative, interventional and surgical methods.

**Case presentations:**

A 67-year-old male was admitted to our institute. He had recurrent chest tightness and chest pain for half a month, which worsened in one day. He was diagnosed with acute non-ST-segment elevation myocardial infarction. Guidewire fracture was caused by improper manipulation during percutaneous coronary intervention. We successfully performed rotational atherectomy to remove the fractured guidewire. His symptoms, and condition improved 6 weeks after the removal of fractured guidewire.

**Conclusion:**

Physicians should have higher requirements for the quality of the guidewires and operation techniques.

## Background

Coronary stenting is a common and effective method for the treatment of coronary heart disease. Fractured guidewires are a rare complication of percutaneous coronary intervention. Fractures occur in association with manipulation and because of the complexity of the coronary artery, and they can cause a series of serious complications, such as myocardial infarction and secondary thrombosis. Common treatments for fractured guidewires include conservative, interventional and surgical methods. This article reports a case of guidewire fracture caused by improper manipulation during percutaneous coronary intervention. We successfully performed rotational atherectomy to remove the fractured guidewire.

## Case presentation

**Fig. 1:**
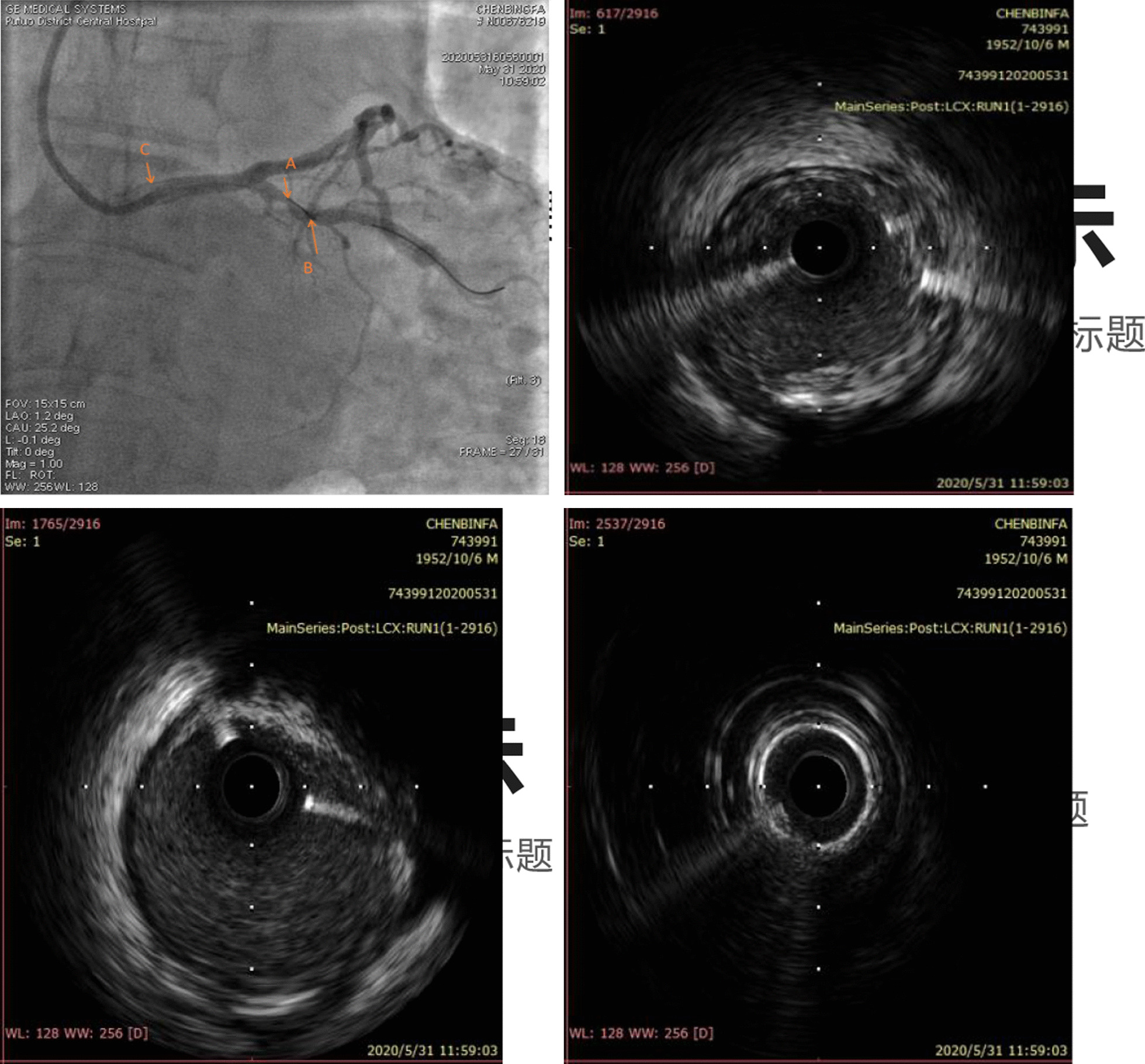
Fractured guidewire in the LCX (A B C). **A** The proximal end of the broken guide wire. **B** The distal end of the broken guide wire. **C** Left main coronary artery opening

**Fig. 2:**
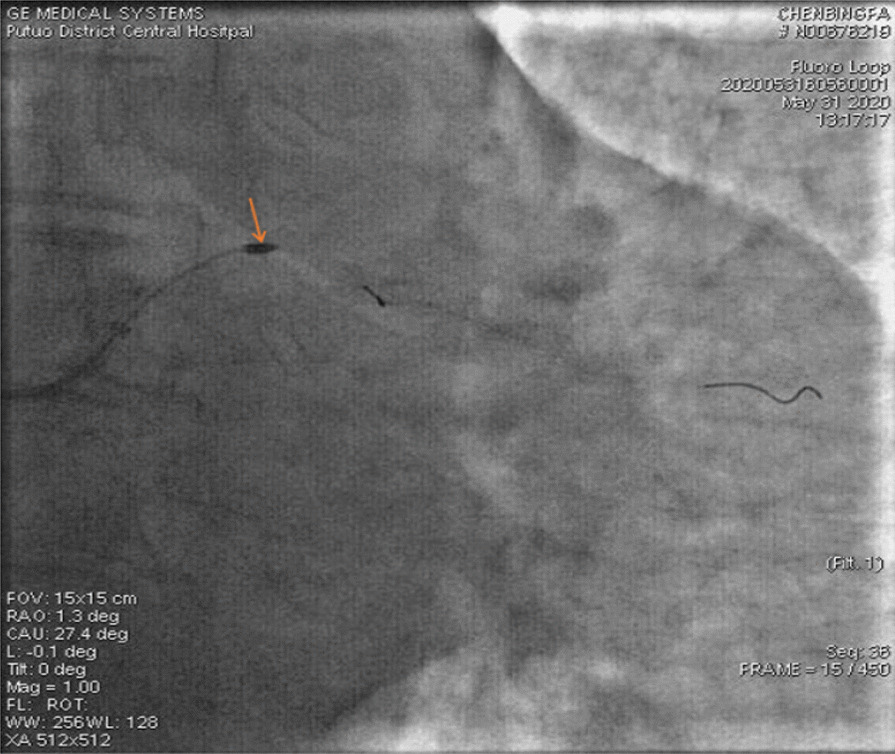
Rotary-grinding guidewire

**Fig. 3:**
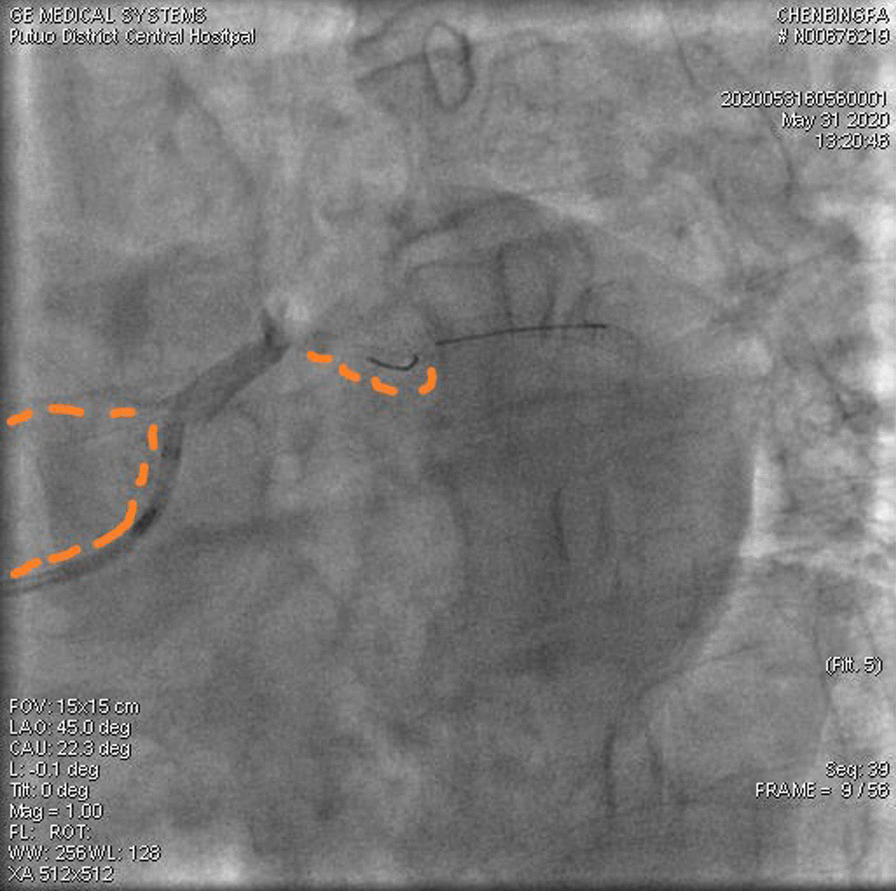
Results of post coronary rotation

**Fig. 4:**
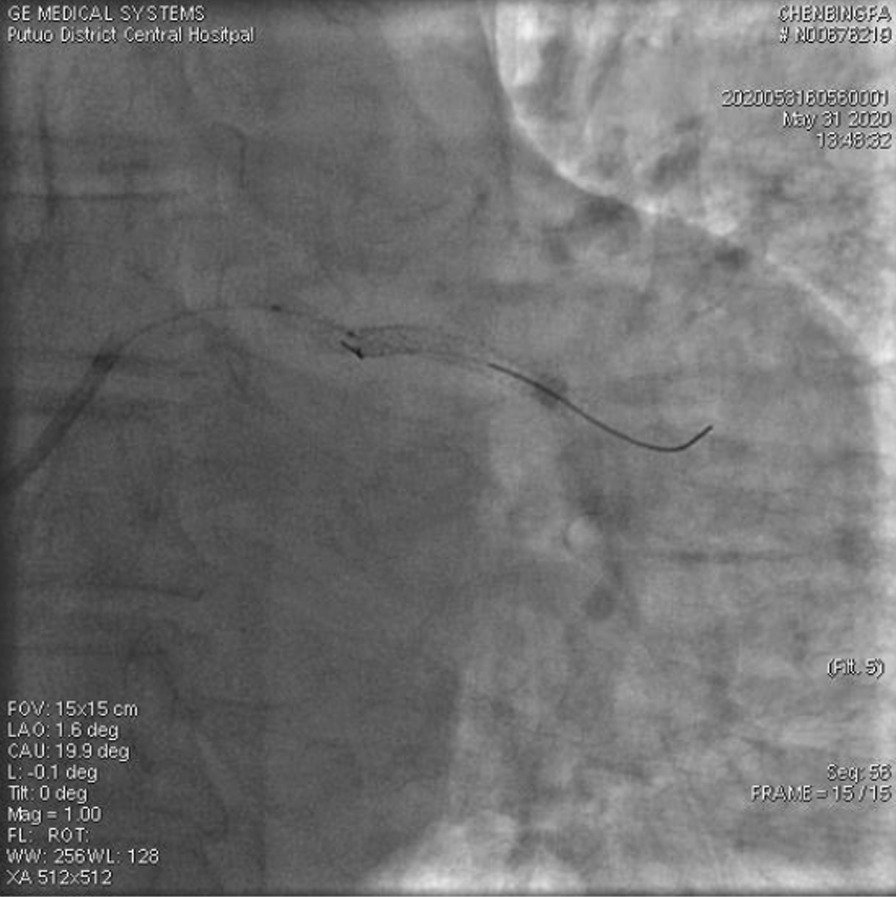
placement of the second stent

**Fig. 5:**
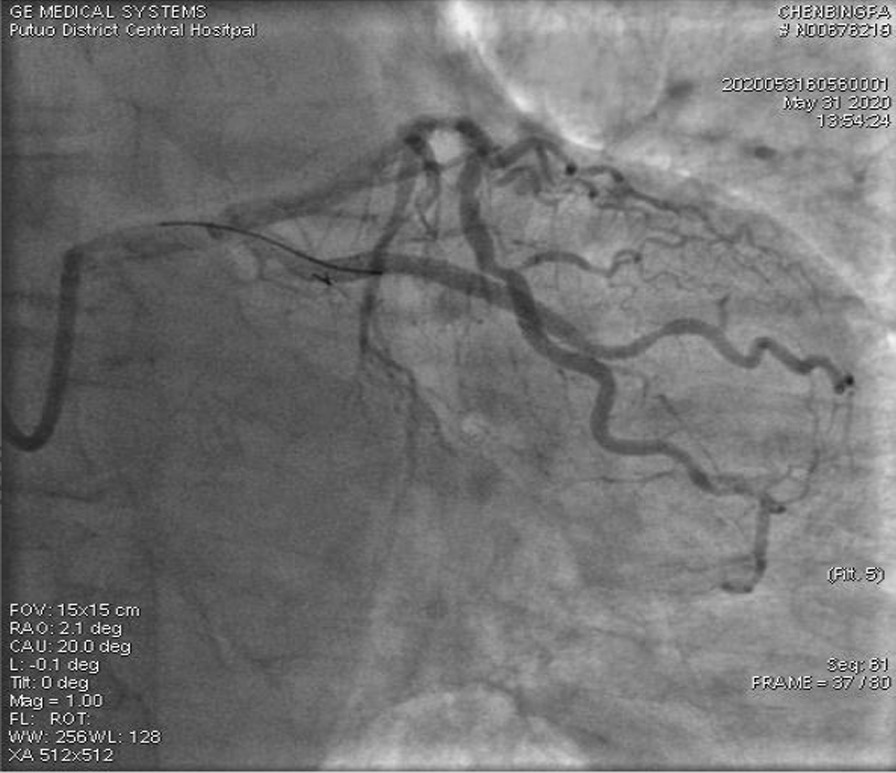
Results of re-examination (postoperative)

A 67-year-old male was admitted to our institute on May 31, 2020. He had recurrent chest tightness and chest pain for half a month, which worsened in one day. Vital Signs were:blood pressure 124/80 mmHg, pulse 75 /min,respirations 22 /min. Electrocardiogram exhibited ST-segment elevation in some leads, Myocardial enzyme profiles were: myoglobin: > 1000 ng/ml, brain natriuretic peptide: 66.88 pg/ml, kinase isoenzyme: 127.58 ng/ml, hypersensitive troponin I: 102.358 ng/ml. He was diagnosed with acute non-ST-segment elevation myocardial infarction. Diagnostic angiography showed that the LCX had 100% stenosis. The LAD had severe stenosis in the proximal and middle portions. The RCA had severe stenosis in the middle portion. Therefore, we directly implanted a 3.0*28 rapamycin-eluting stent in the distal part of the LCX. Dilatation pressure was 11ATM*10 s. Then, we planned to implant another stent. Unfortunately, the stent failed to cross the lesion. We tried to use a Guidezilla catheter to insert a stent. However, the stent still had not passed through the lesion. Then, we decided to insert a double guidewire. When the guidewire passed through the distal end of the stent, it inserted into the stent and hooked on its edge. The guidewire was not successfully withdrawn and broke. IVUS showed that the broken end of the guidewire was 40 mm inside the guiding catheter (Fig. 1). We decided to insert a rotational catheter to cut the guidewire in the LCX so that we could remove the guidewire. Therefore, we implanted a 1.5 mm rotational catheter at 144,000 rpm*18 s. Angiography indicated that the guidewire was successfully divided into two parts. One part was in the guiding catheter (which is usually in the main coronary artery), and the other part was in the LCX (Fig. 2). We used the balloon to squeeze the guidewire in the guiding catheter. Then, we removed the guidewire together with the guiding catheter. After that, we placed the catheter under IVUS guidance again to confirm the position of the broken end of the guidewire in the LCX (Fig. 3). We implanted a 3.5*20 rapamycin-eluting stent to link it with the original stent (Fig. 4). The expansion pressure was 8 ATM*10 s. The remaining guidewire was covered by the stent. In the end, the LCX had no stenosis. The treated coronary arteries had TIMI grade 3 blood flow, and there were no parts of the guidewire in the stent (Fig. 5). He received aspirin 100 mg/day and clopidogrel 75 mg/day as double antiplatelet therapy after PCI.

**Fig. 6:**
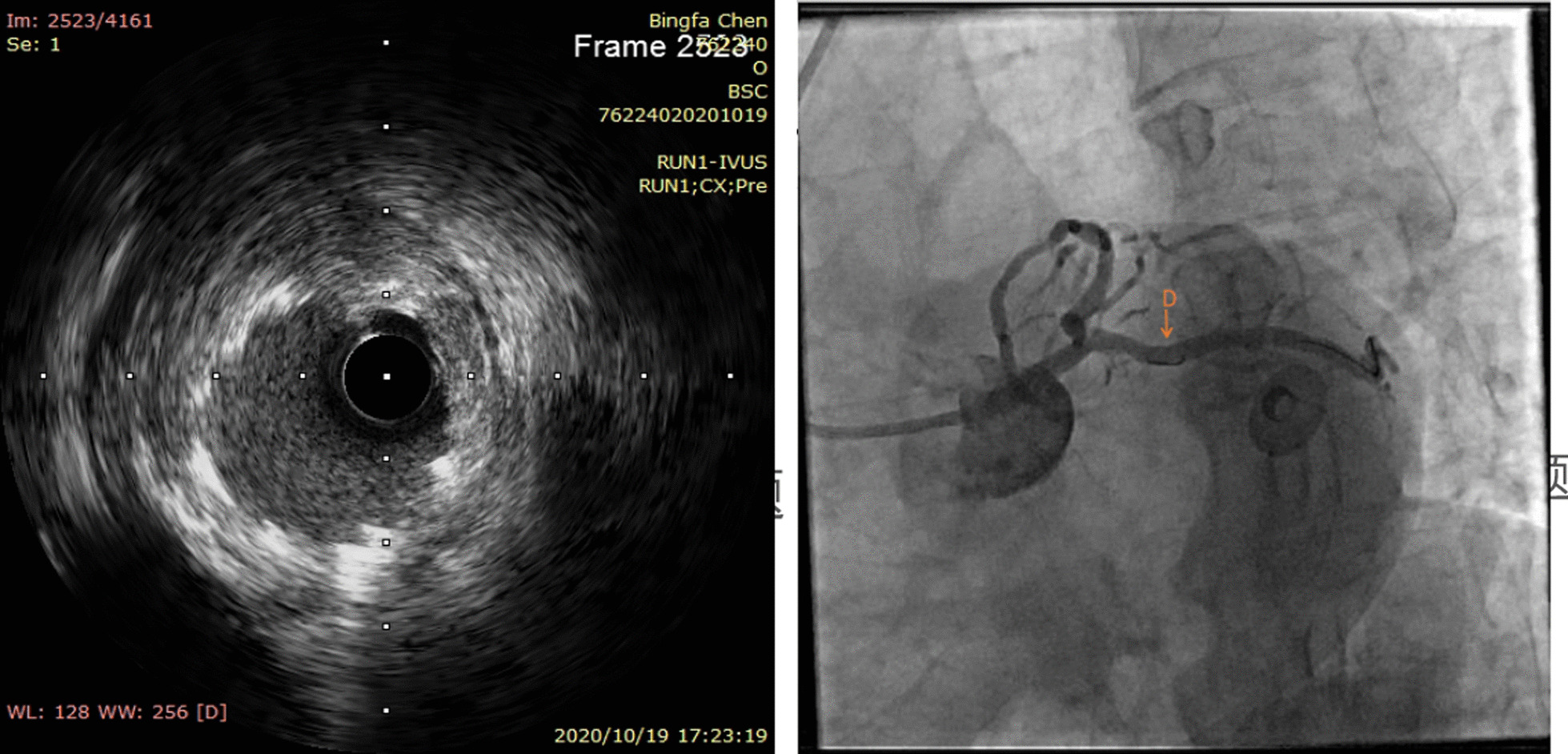
Follow-up results of re-examination (6 months later) ivus

Six months later, the patient was admitted to the hospital for re-examination. Coronary angiography indicated that there was no restenosis in the stent. IVUS showed that the stent adhered well, and high-density shadows (indicating the broken guidewire) were visible between the stent and blood vessel. We implanted a 3.0*38 rapamycin-eluting stent in the distal part of the RCA. Dilatation pressure was 14ATM*10 s. The patient had no chest tightness or shortness of breath (Fig. 6).

## Discussion and conclusions

The overall incidence of guidewire fracture ranges from 0.1 to 0.3% [1]. Therefore, it is very rare during percutaneous coronary interventions (PCIs). Fracture occurrence may be related to vascular anatomy. Excessive rotation, entry, and withdrawal of the guidewire may lead to the wire getting stuck in calcified complex coronary vessels [2]. Broken guidewires can cause serious complications, such as myocardial infarction, coronary artery perforation, cardiac tamponade, and thrombosis [3].

The common options for removing fractured guidewires include conservative, interventional and surgical treatments. In cases where a small part of the guidewire is broken and the patient is asymptomatic conservative treatments should be considered, and the guidewire can be left in the original position. In addition, these patients should take dual antiplatelet therapy with a follow-up period of a half year. Some experts consider that the long-term prognosis of patients with fractured guidewires in the coronary artery is good [4]. There is no special treatment for this condition. Interventional treatment includes stent placement and the use of multiple circumvoluted guidewires. Stent placemen is a common treatment for broken guidewires. Before using this method, the length of the stent must be clear. The stent can completely cover the broken guidewire. When the broken guidewire enters the aorta or a location that the stent cannot reach, the stent could cause thrombosis and aggravate the condition [5]. When the guidewire is broken in small or medium blood vessels, multiple circumvoluted guidewires can be used to move the broken guidewire back into the catheter [6]. If the broken guide wire cannot be removed by a traditional interventional method, there are various coronary snares, such as the EN intravascular net catcher, that can be used. It is composed of three cable metal rings and can firmly grasp and safely remove the broken guidewire. The platinum wire wrapped around each ring can enhance the angiographic effect [7]. If the patient worsens and develops hemodynamic instability, surgical treatment should be considered to completely remove the guidewire. Revascularization is then performed through coronary artery bypass grafting. However, surgical treatment has a high risk of bleeding and a long recovery time [8].

In this case, the three coronary vessels had severe stenosis. First, the stent and guidewire were successfully passed through the coronary artery. When we advanced and withdrew the guidewire, the front point of the guidewire caught on the stent. As a result, the coil of the guidewire was excessively straightened and it broke. In this case, the broken section of the guidewire was not in the coronary artery, so the problem could not be simply solved by stent coverage. The broken section of the guidewire was 40 mm away from the ostium. If covered by a stent, a 40 mm guidewire section could permanently remain in the aorta. Moreover, the cross-main stent would inevitably affect the opening of the LAD. This could make interventional treatment difficult in the future. When the snare pulls on the guidewire, this may further increase the deformation of the original stent due to traction and increase the risk of thrombosis. Therefore, we used rotational atherectomy in this case. The guidewire outside the LCX was removed while the guidewire in the LCX was covered by a stent. The results of follow-up suggest that this method is safe and feasible.

The common complications of rotational atherectomy include coronary artery spasm, coronary artery dissection, rotational head incarceration, the slow-flow/no-reflow phenomenon and perforation [9]. We should have higher requirements for rotational atherectomy. The operators adopt a smaller rotational head and control the rotational atherectomy time. We gently push the rotary grinding head during operation to avoid a sudden drop in rotational speed and vibration. In addition, we pay attention to the patient's heart rate, blood pressure and ST-segment changes So we can reduce the complications and make the operation safer and more effective.

In short, guidewire rupture is a serious complication during percutaneous coronary interventions. Although it is very rare, it should be well recognized. In particular, patients with twisting blood vessels should be given more attention for this complication. The coronary artery characteristics and clinical conditions should be taken into consideration to select the best personalized treatment plan. Taking these factors into consideration can reduce the risk of bleeding and death, improving the survival rate of patients. At the same time, we should have higher requirements for the quality of the guidewires and operation technique.

## Data Availability

The datasets used and/or analysed during the current study are available from the corresponding author on reasonable request.

## Abbreviations

PCI: Percutaneous coronary intervention
LCX: Left circumflex
LAD: Left anterior descending artery
